# The Alzheimer's disease plasma proteome

**DOI:** 10.1002/alz70856_106618

**Published:** 2026-01-10

**Authors:** Lavanya Jain, Maria Khrestian, Elizabeth D. Tuason, James B Leverenz, Lynn M Bekris

**Affiliations:** ^1^ Cleveland Clinic Genomic Medicine Institute, Cleveland, OH, USA; ^2^ Cleveland Clinic, Cleveland, OH, USA; ^3^ University of Washington, Seattle, WA, USA

## Abstract

**Background:**

There is growing evidence supporting a relationship between the plasma proteome and AD‐related pathology. However, less is known about sex differences in the AD plasma proteome or the relationship with AD‐related pathology. We hypothesize that the plasma proteome is different in AD compared to other clinically assessed groups, such as, cognitively normal individuals (CN), mild cognitive impairment (MCI), dementia with Lewy bodies (DLB) or Parkinson's Disease (PD) as well as by sex or underlying cerebrospinal fluid (CSF) pTau_181_/Aβ_42_ status within these clinical groups.

**Methods:**

Plasma samples from CN (*n* = 56), MCI (n60), AD (*n* = 80), DLB (*n* = 32), and PD (*n* = 22) were obtained from existing biobanking resources: (1) The Cleveland Clinic Lou Ruvo Center for Brain Health Aging and Neurodegeneration Biobank, (2) Cleveland Alzheimer's Disease Research Center and (3) the Dementia with Lewy Bodies Consortium. The SomaLogic SomaScan 11K Assay v5.0 was used to evaluate the plasma proteome to quantify 11,000 protein measurements simultaneously. Significant proteins by group comparison, while adjusting for covariates, were selected as biomarker candidates of interest. These biomarker candidates were then evaluated by receiver operating characteristic (ROC) significance.

**Results:**

Our preliminary data indicates that multiple proteins were significantly different between groups suggesting that a plasma biomarker signature can differentiate AD/ADRD. Several were both significantly different by group and by ROC for group comparisons (e.g. CN vs AD: CCL15, REN, NPTXR). When comparing AD and DLB, many plasma biomarker candidates had significant Area Under the Curve (AUC) and Youden Index. One biomarker candidate had an AUC of >0.80 for comparisons with PD (MCI vs PD, AD vs PD: TUBA1A). Several plasma biomarker candidates were significantly different by sex and CSF pTau_181_/Aβ_42_status.

**Conclusion:**

These preliminary findings warrant further study of the plasma proteome in AD and is a first step in defining a blood‐based biomarker tool for detection of underlying pathobiological features that are AD/ADRD specific. This will help guide precision medicine therapeutic strategies in AD/ADRD.

